# Examine the Availability and Safety of Mucosal Cutting Biopsy Technique for Diagnosis of Gastric Submucosal Tumor

**DOI:** 10.1155/2019/3121695

**Published:** 2019-05-02

**Authors:** Kazuhiro Mizukami, Osamu Matsunari, Ryo Ogawa, Yuka Hirashita, Kazuhisa Okamoto, Kensuke Fukuda, Akira Sonoda, Hidetoshi Akiyama, Sotaro Ozaka, Yoshinari Kawahara, Tadayoshi Okimoto, Masaaki Kodama, Kazunari Murakami

**Affiliations:** Department of Gastroenterology, Oita University, 1-1, Idaigaoka, Hasama, Yufu, Oita 879-5593, Japan

## Abstract

**Objectives:**

Differentiating gastrointestinal stromal tumor (GIST) from other submucosal tumors (SMTs) is important in diagnosing SMT. GIST is an immunohistological diagnosis that cannot be made from images alone. Tissue sampling of tumor sites is thus becoming increasingly important. In this study, the utility and associated complications of mucosal cutting biopsy (MCB) for gastric SMTs were investigated.

**Methods:**

This was a case series study. The subjects were patients aged ≥20 years old in whom an SMT was seen on esophagogastroduodenography and who underwent MCB between January 2012 and December 2016. Patient information, endoscopy findings, gastric SMT size, pathological diagnosis, and other information were gathered from medical records. The SMT size was the maximum diameter that could be visualized on EUS. The pathological diagnosis was made with hematoxylin-eosin staining, with immunostaining added to diagnose GIST. The endpoint was the histopathological diagnostic yield. Risk assessment using the Miettinen classification and modified Fletcher classification was also done for GISTs treated with surgery.

**Results:**

The mean tumor diameter was 15.4 mm. The tumor diameter was ≥20 mm in seven patients and <20 mm in 23 patients. The tissue-acquiring rate was 93.3%. A histological diagnosis could not be made in two patients. The only complication was that bleeding required endoscopic hemostasis during the procedure in one patient, but no subsequent bleeding or no postoperative bleeding was seen.

**Conclusions:**

MCB is an appropriate and safe procedure in the diagnosis of gastric SMTs. Many hospitals will be able to perform MCB if they have the environment, including skills and equipment, to perform endoscopic submucosal dissection.

## 1. Introduction

A submucosal tumor (SMT) is defined as a tumor that develops in a layer beneath the mucosa in the gastrointestinal wall [1]. The incidence of SMTs in the gastrointestinal tract is not low, with that of gastric submucosal tumors discovered during esophagogastroduodenography considered to be about 0.4% [2]. Most SMTs have been thought to be benign leiomyomas, and in nearly all cases, a watchful waiting approach has been adopted. However, the disease concept of gastrointestinal stromal tumor (GIST), a potentially malignant tumor, has been established with advances in immunohistological techniques, and this has transformed the clinical approach to SMTs [3, 4]. It has been shown that c-kit gene mutation is present in about 90% of GISTs, which are potentially malignant; that metastasis is seen even with small GISTs; and that those of 2 cm or less in the stomach are curable if they are locally resected [3–6]. The National Comprehensive Cancer Network guidelines in the United States and the European Society for Medical Oncology guidelines in Europe were revised in 2004, followed by the GIST treatment guidelines of the Japan Society of Clinical Oncology in Japan [4, 5, 7]. GIST is now classified as a potentially malignant tumor, and the first-line treatment for resectable GIST, regardless of size, is local surgical treatment.

Consequently, differentiating GIST from other SMTs is important in diagnosing SMT today. GIST is an immunohistological diagnosis; it cannot be diagnosed from images alone. Tissue sampling of tumor sites is thus becoming increasingly important.

Since SMTs exist submucosally, tumor tissue sampling with regular forceps biopsy presents many difficulties. In the current, third edition of the GIST treatment guidelines of the Japan Society of Clinical Oncology, endoscopic ultrasound-guided fine needle aspiration biopsy (EUS-FNAB), in which the biopsy needle is inserted and tissue is sampled under endoscopic ultrasound (EUS) guidance, is considered to be the most effective. At the same time, a special endoscope device, expert technique, and the presence of a pathologist or cytologist to confirm whether the collected specimen is appropriate tumor tissue are needed, leading to the problem that the test cannot be easily done in ordinary hospitals [5].

Mucosal cutting biopsy (MCB) often involves endoscopic submucosal dissection (ESD), which is a common technique today. In MCB, the lesion is biopsied under direct vision by cutting the gastric mucosa with an electric knife and sufficiently exposing the SMT [8]. It can be done if an electrosurgical unit is available, so it is a procedure that can be performed at many institutions. In this study, MCB was performed for gastric SMTs, and its utility and associated complications were investigated.

## 2. Methods

This was a case series study. The subjects were patients aged ≥20 years old in whom an SMT was seen on esophagogastroduodenography and who physicians diagnosed as adaptable for MCB between January 2012 and December 2016. Patients with a bleeding tendency, those who were taking antithrombotic drugs, those whose SMT was outside the gastric wall, and those whose general condition was poor were excluded. For patients after January 2014, consent was obtained in writing. For patients before that time, an announcement of the intent to use test information and results was posted on the web page of the Department of Gastroenterology, Oita University, and an opportunity was provided for patients to refuse to allow their information to be used.

**Figure 1:**
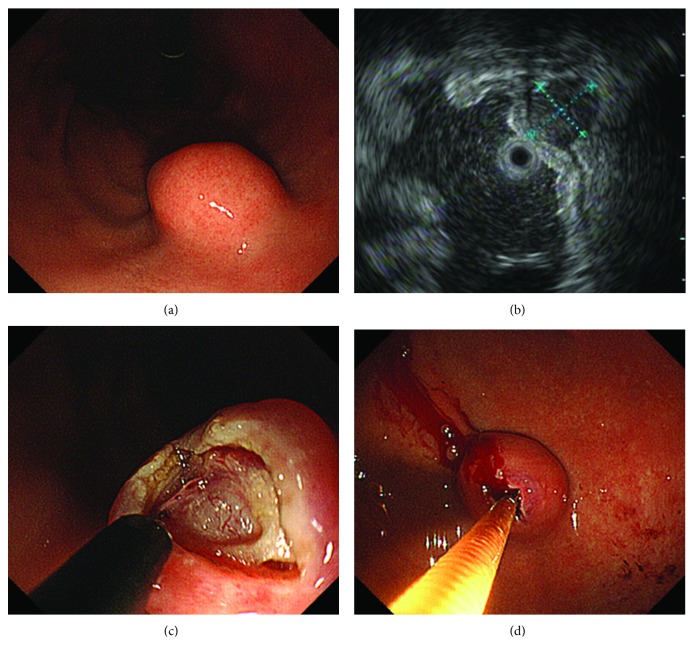
Mucosal cutting biopsy technique. (a, b) Using EGD and EUS, it was confirmed that the tumor was an SMT, that the tumor did not protrude outside the gastric wall, and that the tumor was solid, not cystic or vascular. (c) An incision of ≥5 mm was made in the superficial mucosa of an elevated area of the tumor with a needle knife, and the tumor was adequately exposed. (d) A sample was taken directly using biopsy forceps.

MCB was performed with the following method (Figure 1): (1) Using EUS, it was confirmed that the tumor was an SMT, that the tumor did not protrude outside the gastric wall, and that the tumor was solid, not cystic or vascular. (2) Exposures of ≧5 mm and ≦10 mm were made in the superficial mucosa of an elevated area of the tumor with a needle knife. (3) The tumor was adequately exposed using FORCED COAG mode. (4) Samples were taken directly using biopsy forceps about 5-7 times. (5) Hemostatic procedures were performed if there was bleeding after the sample was taken. MCB was performed by 3 endoscopists (K.M., O.M., and R.O.). All of them have experiences of more than 100 cases in ESD.

We employed a GIF-Q260J endoscope equipped with a water-jet system (Olympus Medical Systems Co., Tokyo, Japan), a VIO 200 D (ERBE Elektromedizin GmbH, Tübingen, Germany), and a needle knife (KD-1L; Olympus Medical Systems Co.). The mucosal incision was done with the ENDO CUT mode (effect 2, duration 2, and interval 3), and the tumor was exposed with the FORCED COAG mode (effect 3, 40 W). The procedure was principally done on an outpatient basis. Afterward, proton pump inhibitor and ecabet sodium hydrate, which has a gastric mucosal coating action, were prescribed.

Patient information, endoscopy findings, gastric SMT size, EUS findings, pathological diagnosis, and other information were gathered from medical records. The SMT size was the maximum diameter that could be visualized on EUS. The pathological diagnosis was made with hematoxylin-eosin staining, with immunostaining (CD34, c-kit, alpha-smooth muscle actin (a-SMA), S-100 protein, and Desmin) added for a diagnosis of GIST.

The endpoint was the histopathological diagnostic yield. Risk assessments using the Miettinen classification and the modified Fletcher classification were also done for GISTs treated with surgery [9, 10].

This study was performed in compliance with the Declaration of Helsinki and the ethical guidelines for medical and health research involving human subjects, and it was conducted after receiving an approval for the protocol and informed consent form from the General Clinical Research Center, Oita University Hospital (UMIN-CTR ID: UMIN000012800).

## 3. Results

**Figure 2:**
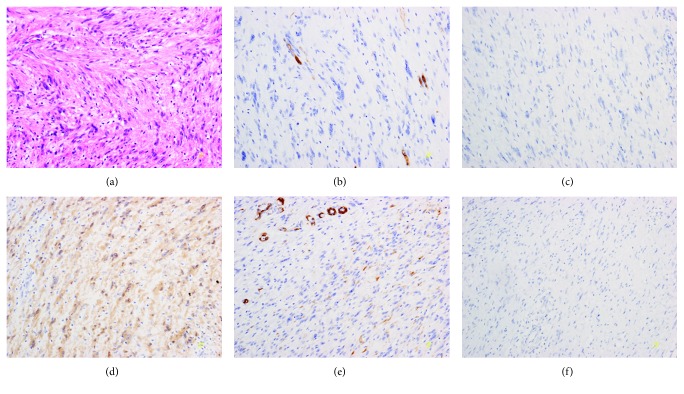
Pathologic findings of resected specimen in Case 22 (diagnosis is schwannoma). (a) Tissue was composed of broad bundles of elongated cells (hematoxylin-eosin stain, ×200). Immunohistochemistry staining for (b) CD34, (c) c-kit, (e) alpha-smooth muscle actin (a-SMA), and (f) desmin are negative. (d) The tumor strongly stains for the S-100 protein.

The characteristics of the 30 patients are summarized in Table 1. The patients had an average age of 61.0 ± 18.4 years (range, 35-85 years) and comprised 14 men and 16 women. The mean tumor diameter was 15.4 mm. The tumor diameter was ≥20 mm in seven patients and <20 mm in 23 patients. The tumor site was the fornix and cardia of stomach in nine patients, the body of stomach in 16 patients, and the angle and antrum in five patients. The tissue-acquiring rate was 93.3%. A histological diagnosis could not be made in two patients. One patient was initially diagnosed with a schwannoma, but in re-examination six months later, this patient was diagnosed with GIST and underwent a surgical procedure (but was eventually diagnosed as schwannoma in Case 22) (Figure 2). In one patient, the tumor was <20 mm, and after consultation with the patient, a watchful waiting strategy was adopted (Case 13).

Of the 14 patients diagnosed with GIST, surgery was performed at the authors' hospital for 11 patients. The GIST risk classification for these patients was very low risk or none in the Miettinen classification and low risk or very low risk in the modified Fletcher classification.

The only complication was that bleeding required endoscopic hemostasis during the procedure in one patient (Case 19), but no subsequent bleeding or no postoperative bleeding was seen.

## 4. Discussion

In diagnosing SMT, the tumor is seldom exposed on the mucosal surface, and the diagnosis is frequently difficult. With the appearance of EUS-FNAB, the diagnosis of SMT has progressed dramatically. In the GIST treatment guidelines, EUS-FNAB is recommended as the sole effective method [5]. The diagnostic accuracy of EUS-FNAB is 82-91% [11–13]. Many studies are currently being conducted to improve the diagnostic yield, and the diagnostic yield of EUS-FNAB is expected to increase in the future with the appearance of forward-viewing linear echoendoscopes [14, 15], improvements to the technique [16–18], and the introduction of rapid on-site cytological evaluation [19].

Measures for GIST, which account for a high percentage of SMTs, are also needed. In the Japanese GIST treatment guidelines, the indication for EUS-FNAB is SMT ≥ 20 mm [5]. However, there have also been reports that the risk of malignancy is high even in GISTs of under 20 mm. Akahoshi et al. reported that, of 43 patients who underwent surgery for GISTs of less than 20 mm, 23% corresponded to medium risk in the modified Fletcher classification for GIST [20]. Aso et al. reported that liver metastasis occurred postoperatively in a patient with a gastric GIST of 15 mm [21]. Hence, for SMT, it is important to conduct a careful examination with EUS or other techniques to the maximum extent possible even for small tumors and to actively perform histopathological evaluation if GIST is suspected.

The MCB investigated in the present study has the following advantages. The mucosal incision is a skill that we endoscopists have already acquired in ESD, and it does not require learning to use the special scopes or specific techniques used in EUS and EUS-FNAB. Other advantages are that the tumor is plainly exposed and biopsy samples are easily obtained, samples can be collected in sufficient amounts since they are collected with biopsy forceps, and investment to develop the setting, such as purchasing new equipment, is not necessary [22]. All of the tumors in the present investigation were relatively small, including tumors in eight patients that were 10 mm or less. However, because the tumors were clearly visible when samples were obtained, a pathological diagnosis could be made in almost all cases and the treatment strategy determined. Kataoka et al. performed MCB for SMT in 18 patients, from which a histological diagnosis was established in all cases and no posttest complications were seen [8]. Kobara et al. reported a mucosal incision method in which a small incision is made in the mucosa, a tunnel to the submucosal layers is formed, and the SMT is biopsied with forceps [23]. Other methods such as using a snare simultaneously to expose mucosa have also been reported, and they are also achieving good results [24]. Thus, MCB is thought to have a high diagnostic yield because the tumor is visually recognized and biopsied.

In the present study, a sample could not be obtained in one patient. Kataoka et al. collected samples after injecting physiological saline into the mucosa, making a large incision in the mucosal surface, and expanding the visual field [8]. In the present study, we conducted a smaller incision compared to the previously reported study [8, 22]. As a result, however, the collected samples were insufficient in some cases. Establishing an appropriate MCB procedure using the experience of multiple institutions will be important.

Bleeding that required hemostatic measures during MCB was seen in only one of the present patients. Another strength of this procedure is that the hemostatic measures are familiar, since the procedure is the same as with ESD. However, there are no past reports of subsequent bleeding, and we find that biopsy procedure including MCB which exposes mucosa can be performed safely. [8, 22–24].

This study has several limitations. One is that it was a single-institution study with a small sample size. Another is that, since it was a case series study intended as a pilot study, some cases were registered retrospectively and others were also registered by the physician's decision. In the future, our goal is to enroll many patients prospectively and establish MCB together with EUS-FNAB as a useful test in the treatment of SMT.

## 5. Conclusions

MCB is an appropriate and safe procedure in the diagnosis of gastric SMT. Many hospitals will be able to perform MCB if they have the environment, including skills and equipment, to perform ESD.

## Figures and Tables

**Table 1:** Endoscopic findings and pathological diagnosis of gastric SMT patient performed with mucosal cutting biopsy.

Patient			Information of SMT by EGD, EUS, and MCB				Diagnosis by resected specimen					
Case	Age	Gender	Location	Size (mm)	Diagnosis	Ki-67 (%)	Definitive diagnosis	Size (mm)	Nuclear fission	Ki-67 (%)	Miettinen classification	Modified Fletcher classification
1	76	M	Body	28	GIST	<5	GIST	35	<5	1.8	Very low	Low
2	74	M	Body	19	Heterotopic pancreas							
3	46	M	Antrum	8	Heterotopic pancreas							
4	56	M	Fornix	17	GIST	6.6	GIST	23	<5	—	Very low	Low
5	82	M	Fornix	22	GIST		GIST	30	<5	4	Very low	Low
6	53	F	Angle	13	Heterotopic pancreas							
7	74	F	Body	12	GIST	2.2	GIST	18	2	2	Very low	Low
8	42	M	Cardia	24	Leiomyoma							
9	73	F	Angle	15	Heterotopic pancreas							
10	35	F	Body	13	Heterotopic pancreas							
11	68	F	Body	26	GIST	0	GIST	30	<5	2.9	Very low	Low
12	66	F	Cardia	14	GIST	1	GIST	35	<5	3.6	Very low	Low
13	62	M	Angle	12	Negative							
14	70	F	Cardia	13	Leiomyoma							
15	65	M	Body	23	GIST	2.22	GIST	40	<5	<2	Very low	Low
16	72	F	Cardia	15	GIST	0						
17	69	M	Body	29	GIST	4.3						
18	45	M	Body	29	GIST	0	GIST	25	<5	<2	Very low	Low
19	73	F	Body	9	Heterotopic pancreas							
20	44	F	Body	8	Schwannoma							
21	38	M	Body	17	Heterotopic pancreas							
22	45	F	Body	10	GIST	0	Schwannoma	10	0	<2	—	—
23	82	F	Body	8	GIST	0						
24	59	F	Cardia	18	Leiomyoma							
25	85	M	Cardia	15	GIST	6.5	GIST	15	<5	<2	None	Very low
26	72	M	Body	9	Leiomyoma							
27	70	F	Fornix	9	GIST	Unknown	GIST	10	<5	<2	None	Very low
28	57	F	Antrum	8	Heterotopic pancreas							
29	35	M	Body	11	Leiomyoma							
30	42	F	Body	8	Leiomyoma							

SMT: submucosal tumor; EGD: esophagogastroduodenography; EUS: endoscopic ultrasound; MCB: mucosal cutting biopsy; GIST: gastrointestinal stromal tumor.

## Data Availability

All the data used to support the findings of this study are included within the article.
